# Supplementary material for: Development of a Novel Anti-CD44 Variant 8 Monoclonal Antibody C44Mab-94 against Gastric Carcinomas
Source: Antibodies (Basel). 2023 Jul 4;12(3):45. doi: 10.3390/antib12030045 (PMC10366929; doi:10.3390/antib12030045)
Supplement: Supplementary file 1 [file antibodies-12-00045-s001.zip › Supplementary Figs S1-3.pdf]

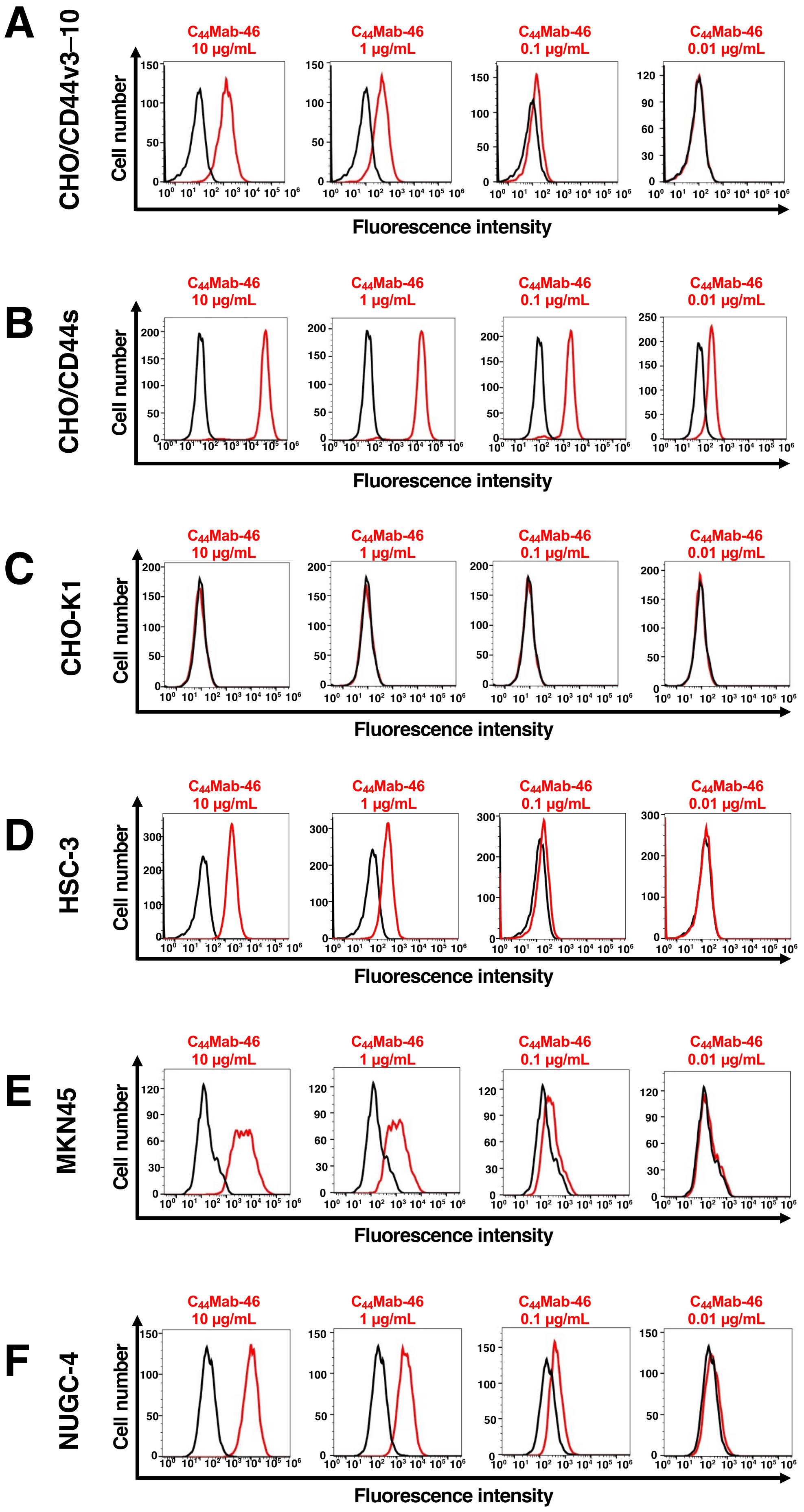

Figure S1 Recognition of CHO/CD44s, CHO/CD44v3–10, and cancer cell lines by C_{44}Mab-46 using flow cytometry. CHO/CD44v3–10 (**A**), CHO/CD44s (**B**), CHO-K1 (**C**), HSC-3 (**D**), MKN45 (**E**), and NUGC-4 (**F**) were treated with 0.01-10 μg/mL of C_{44}Mab-46, followed by treatment with Alexa Fluor 488-conjugated anti-mouse IgG (Red line). The black line represents the negative control (blocking buffer).

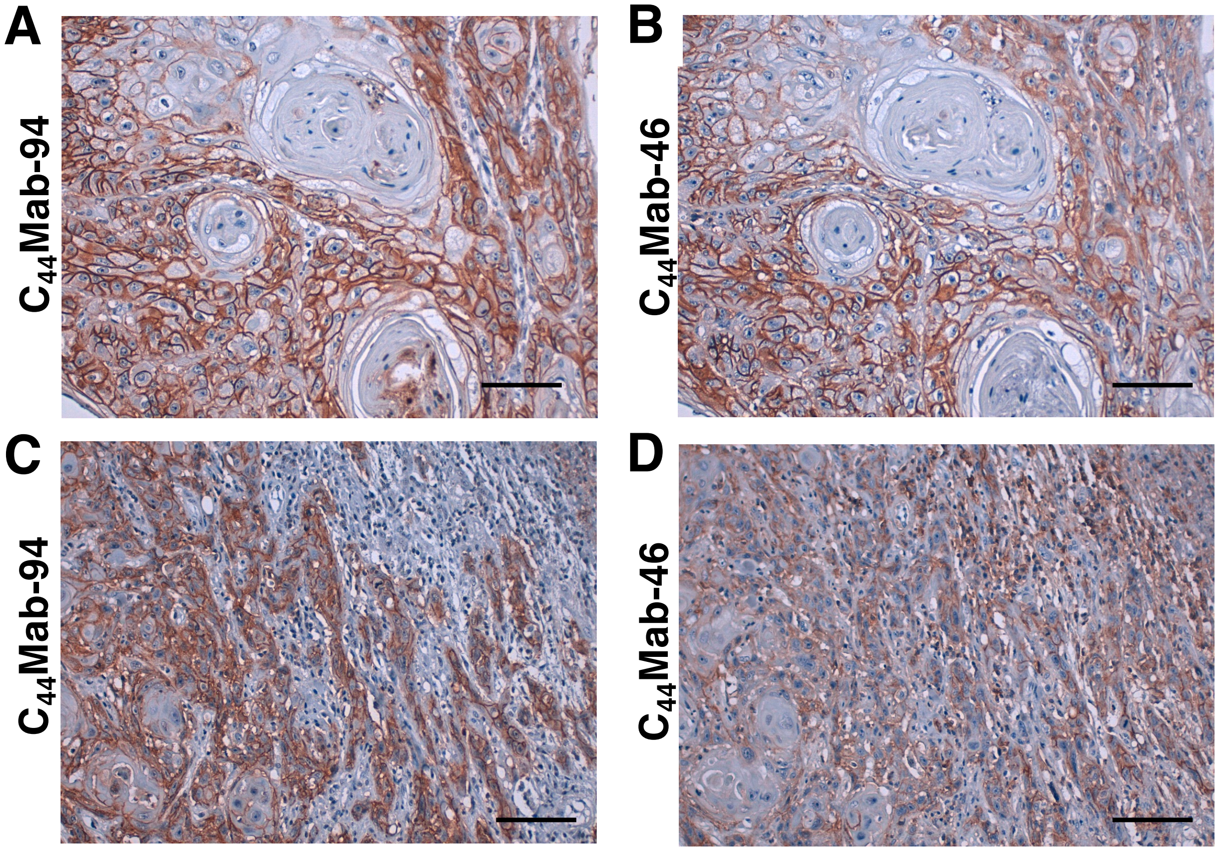

Figure S2. Immunohistochemistry using C_{44}Mab-94 and C_{44}Mab-46 against OSCC. (**A**–**D**) After antigen retrieval, serial sections of OSCC tissue array (OR601c). were incubated with 5 μg/mL of C_{44}Mab-94 or 5 μg/mL of C_{44}Mab-46, followed by treatment with the Envision+ kit. The chromogenic reaction was performed using 3,3′-diaminobenzidine tetrahydrochloride (DAB), and the sections were counterstained with hematoxylin. Scale bar = 100 μm.

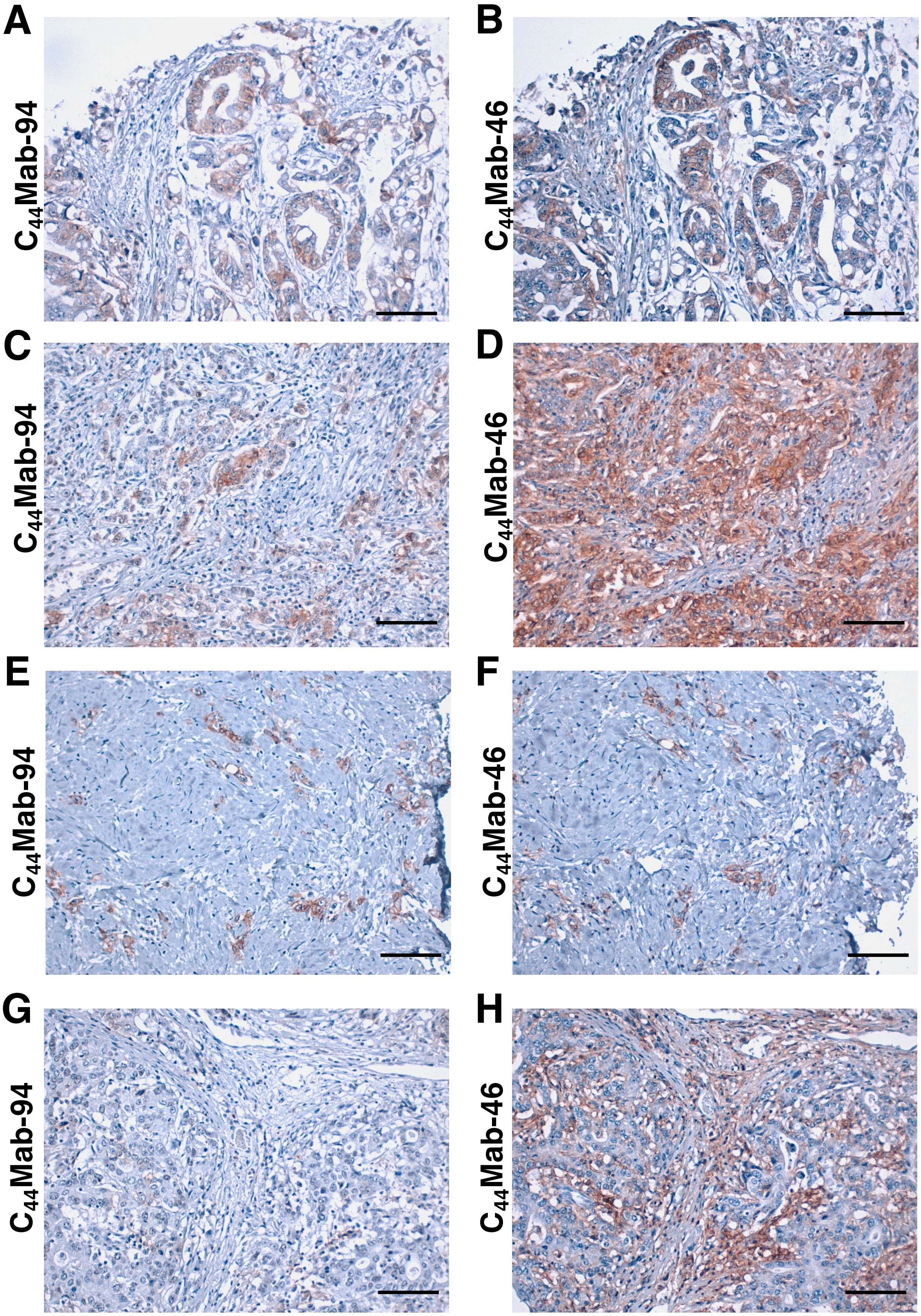

Figure S3. Immunohistochemistry using C_{44}Mab-94 and C_{44}Mab-46 against GC. (**A**–**H**) After antigen retrieval, serial sections of GC tissue array (BS01012e). were incubated with 5 μg/mL of C_{44}Mab-94 or 5 μg/mL of C_{44}Mab-46, followed by treatment with the Envision+ kit. The chromogenic reaction was performed using 3,3′-diaminobenzidine tetrahydrochloride (DAB), and the sections were counterstained with hematoxylin. Scale bar = 100 μm.
