# Supplementary material for: Development of a Novel Anti-CD44 Variant 8 Monoclonal Antibody C44Mab-94 against Gastric Carcinomas
Source: Antibodies (Basel). 2023 Jul 4;12(3):45. doi: 10.3390/antib12030045 (PMC10366929; doi:10.3390/antib12030045)
Supplement: Supplementary file 1 [file antibodies-12-00045-s001.zip › Supplementary Table S1 and S2.pdf]

Table S1, Immunohistochemical analysis using C_{44}Mab-94 and C_{44}Mab-46 against OSCC tissue array.

No.	Age	Sex	Organ	Pathology diagnosis	TNM	C_{44}Mab-94	C_{44}Mab-46
1	78	M	Tongue	SCC of tongue	T2N0M0	++	+
2	40	M	Tongue	SCC of tongue	T2N0M0	++	++
3	75	F	Tongue	SCC of tongue	T2N0M0	++	+
4	35	F	Tongue	SCC of tongue	T2N0M0	+++	++
5	61	M	Tongue	SCC of tongue	T2N0M0	+++	++
6	41	F	Tongue	SCC of tongue	T2N0M0	+++	+
7	64	M	Tongue	SCC of right tongue	T2N2M0	+++	+
8	76	M	Tongue	SCC of tongue	T1N0M0	+++	+
9	50	F	Tongue	SCC of tongue	T2N0M0	+++	++
10	44	M	Tongue	SCC of tongue	T2N1M0	+++	++
11	53	F	Tongue	SCC of tongue	T1N0M0	-	++
12	46	F	Tongue	SCC of tongue	T2N0M0	+	+
13	50	M	Tongue	SCC of root of tongue	T3N1M0	++	++
14	36	F	Tongue	SCC of tongue	T1N0M0	+++	+++
15	63	F	Tongue	SCC of tongue	T1N0M0	+	+
16	46	M	Tongue	SCC of tongue	T2N0M0	+++	+
17	58	M	Tongue	SCC of tongue	T2N0M0	++	+
18	64	M	Lip	SCC of lower lip	T1N0M0	+	++
19	57	M	Lip	SCC of lower lip	T2N0M0	+	++
20	61	M	Lip	SCC of lower lip	T1N0M0	++	+
21	60	M	Gum	SCC of gum	T3N0M0	++	+
22	60	M	Gum	SCC of gum	T1N0M0	+++	+++
23	69	M	Gum	SCC of upper gum	T3N0M0	+	++
24	53	M	Bucca cavioris	SCC of bucca cavioris	T2N0M0	++	+
25	55	M	Bucca cavioris	SCC of bucca cavioris	T1N0M0	++	+
26	58	M	Tongue	SCC of base of tongue	T1N0M0	+	+
27	63	M	Oral cavity	SCC	T1N0M0	+++	+
28	48	F	Tongue	SCC of tongue	T1N0M0	+++	+
29	80	M	Lip	SCC of lower lip	T1N0M0	+	+++
30	77	M	Tongue	SCC of base of tongue	T2N0M0	+++	++
31	59	M	Tongue	SCC of tongue	T2N0M0	++	+
32	77	F	Tongue	SCC of tongue	T1N0M0	++	+
33	56	M	Tongue	SCC of root of tongue	T2N1M0	++	+

34	60	M	Tongue	SCC of tongue	T2N1M0	+++	++
35	62	M	Tongue	SCC of tongue	T2N0M0	+++	+++
36	67	F	Tongue	SCC of tongue	T2N0M0	+++	+++
37	47	F	Tongue	SCC of tongue	T2N0M0	++	++
38	37	M	Tongue	SCC of tongue	T2N1M0	+	+
39	55	F	Tongue	SCC of tongue	T2N0M0	+++	++
40	56	F	Bucca cavioris	SCC of bucca cavioris	T2N0M0	+	+
41	49	M	Bucca cavioris	SCC of bucca cavioris	T1N0M0	-	-
42	45	M	Bucca cavioris	SCC of bucca cavioris	T2N0M0	-	+
43	42	M	Bucca cavioris	SCC of bucca cavioris	T3N0M0	++	+
44	44	M	Jaw	SCC of right drop jaw	T1N0M0	+++	+++
45	40	F	Tongue	SCC of base of tongue	T2N0M0	-	+++
46	49	M	Bucca cavioris	SCC of bucca cavioris	T1N0M0	+++	++
47	56	F	Tongue	SCC of base of tongue	T2N0M0	-	+
48	42	M	Bucca cavioris	SCC of bucca cavioris	T3N0M0	+++	+++
49	87	F	Face	SCC of left face	T2N0M0	+	+
50	50	M	Gum	SCC of gum	T2N0M0	+	+++

-, No stain; +, Weak intensity; ++, Moderate intensity; +++, Strong intensity.

Table S2, Immunohistochemical analysis using C_{44}Mab-94 and C_{44}Mab-46 against GC tissue array (BS01012e).

**No.**	**Age**	**Sex**	**Pathology diagnosis**	**TNM**	**Grade**	**Stage**	**C_{44}Mab-94**	**C_{44}Mab-46**
1	49	F	Adenocarcinoma	T3N1M0	2	IIB	-	+
2	68	M	Adenocarcinoma	T3N0M0	2	IIA	-	++
3	55	M	Adenocarcinoma (sparse)	T4N2M0	2	IIIB	-	-
4	75	M	Adenocarcinoma	T3N1M0	3	IIB	-	+
5	60	F	Adenocarcinoma	T2N1M0	2	IIA	-*	-*
6	48	F	Adenocarcinoma	T3N2M0	3	IIIA	+	+++
7	66	M	Adenocarcinoma	T3N0M0	2	IIA	-	-*
8	55	F	Adenocarcinoma	T2N0M0	1--2	IB	-	++
9	47	F	Adenocarcinoma	T3N1M0	3	IIB	-	-
10	61	M	Adenocarcinoma	T3N1M0	2	IIB	+	+
11	70	M	Adenocarcinoma	T3N1M0	3	IIB	++	++
12	60	M	Adenocarcinoma	T2N1M0	2	IIA	-	+
13	54	M	Adenocarcinoma	T2N1M0	2	IIA	-	-
14	72	M	Adenocarcinoma	T2N1M0	2	IIA	-	-
15	71	M	Adenocarcinoma	T2N1M0	2	IIA	+	+
16	63	M	Adenocarcinoma	T2N1M0	2	IIA	-*	+
17	66	M	Adenocarcinoma	T1N2M0	2	IIA	+	+
18	53	M	Adenocarcinoma	T3N1N0	3	IIB	-*	+
19	68	F	Adenocarcinoma	T2N1M0	2	IIA	-	-
20	64	F	Adenocarcinoma	T2N1M0	2	IIA	-	+
21	50	F	Adenocarcinoma	T3N0M0	3	IIA	-	+
22	72	M	Adenocarcinoma	T2N0M0	2--3	IB	-	++
23	50	M	Mucinous adenocarcinoma	T3N0M0	3	IIA	-	-
24	69	M	Adenocarcinoma	T2N0M0	3	IB	-	++
25	59	M	Adenocarcinoma	T2N0M0	3	IB	-	-
26	35	M	Adenocarcinoma	T3N0M0	3	IIA	-	+
27	43	M	Adenocarcinoma	T3N1M0	3	IIB	-	+
28	60	M	Mucinous adenocarcinoma	T3N1M0	3	IIB	++	++
29	70	F	Adenocarcinoma	T2N0M0	3	IB	-	-
30	64	M	Adenocarcinoma	T2N1M0	3	IIA	+	-

31	45	M	Adenocarcinoma	T3N1M0	3	IIB	-	-
32	35	F	Adenocarcinoma	T3N2M0	3	IIIA	-	+
33	57	M	Adenocarcinoma	T3N1N0	3	IIB	+	++
34	72	M	Adenocarcinoma	T3N1N0	3	IIB	-*	+
35	68	M	Adenocarcinoma	T3N1N0	3	IIB	+	+++
36	72	F	Adenocarcinoma	T3N0M0	3	IIA	-*	++
37	62	M	Adenocarcinoma	T2N2M0	3	IIB	-	-
38	68	F	Adenocarcinoma	T2N1M0	3	IIA	-	+
39	43	M	Adenocarcinoma	T2N0M0	3	IB	-	+
40	55	M	Adenocarcinoma	T3N2M0	3	IIIA	+	+
41	61	M	Adenocarcinoma	T3N0M0	3	IIA	-	-
42	49	F	Adenocarcinoma	T3N1M0	2	IIB	+	+
43	49	M	Adenocarcinoma	T2N1M0	3	IIA	-	+
44	57	M	Adenocarcinoma	T3N1M0	3	IIB	-	-
45	63	M	Adenocarcinoma	T3N2M0	3	IIIA	-	+
46	59	M	Adenocarcinoma (fibrous tissue and blood vessel)	T3N1M0		IIB	-	-
47	60	M	Adenocarcinoma	T2N1M0	3	IIA	+	+
48	46	F	Adenocarcinoma	T3N1M0	3	IIB	+++	+++
49	50	M	Adenocarcinoma	T2N1M0	3	IIA	++	++
50	63	M	Adenocarcinoma	T2N1M0	3	IIA	-	-
51	37	F	Adenocarcinoma	T2N2M0	3	IIB	-	-
52	45	M	Adenocarcinoma	T2N1M0	3	IIA	-	-
53	34	M	Adenocarcinoma	T2N1M0	3	IIA	-	+
54	54	M	Adenocarcinoma	T3N1M0	3	IIB	-	-
55	64	M	Adenocarcinoma	T2N1M0	3	IIA	+	+
56	40	M	Adenocarcinoma	T3N2M0	3	IIIA	-	-
57	68	M	Mucinous adenocarcinoma	T2N1M0	3	IIA	-	-
58	33	M	Mucinous adenocarcinoma	T3N1M0	3	IIB	-	-
59	70	M	Adenocarcinoma	T2N0M0	3	IB	-	-
60	46	M	Mucinous adenocarcinoma	T3N0M0	3	IIA	-	+
61	55	M	Adenocarcinoma	T3N1M0	3	IIB	-	-

62	58	M	Adenocarcinoma	T2N1M0	3	IIA	-	+
63	24	F	Adenocarcinoma	T3N1M0	3	IIB	+	-
64	35	M	Adenocarcinoma	T3N1M0	3	IIB	-	+
65	47	F	Adenocarcinoma	T3N1M0	3	IIB	+	+++
66	64	M	Adenocarcinoma	T2N1M0	3	IIA	+	+
67	56	M	Adenocarcinoma	T3N2M0	2	IIIA	-	+
68	75	M	Adenocarcinoma	T2N0M0	3	IB	-	+
69	48	M	Adenocarcinoma	T2N0M0	3	IB	-*	+
70	48	M	Adenocarcinoma	T2N0M0	3	IB	-	-
71	74	M	Adenocarcinoma	T2N0M0	3	IB	+	++
72	45	F	Adenocarcinoma	T4N0M1	3	IV	++	++

*Nuclear staining was detected.

-, No stain; +, Weak intensity; ++, Moderate intensity; +++, Strong intensity.
